# Explainable artificial intelligence (XAI) in radiology and nuclear medicine: a literature review

**DOI:** 10.3389/fmed.2023.1180773

**Published:** 2023-05-12

**Authors:** Bart M. de Vries, Gerben J. C. Zwezerijnen, George L. Burchell, Floris H. P. van Velden, Catharina Willemien Menke-van der Houven van Oordt, Ronald Boellaard

**Affiliations:** ^1^Department of Radiology and Nuclear Medicine, Cancer Center Amsterdam, Amsterdam UMC, Vrije Universiteit Amsterdam, Amsterdam, Netherlands; ^2^Medical Library, Vrije Universiteit Amsterdam, Amsterdam, Netherlands; ^3^Department of Radiology, Leiden University Medical Center, Leiden, Netherlands; ^4^Department of Oncology, Cancer Center Amsterdam, Amsterdam UMC, Vrije Universiteit Amsterdam, Amsterdam, Netherlands

**Keywords:** deep learning, explainable artificial intelligence, magnetic resonance (MR) imaging, computed tomography (CT) imaging, positron emission tomography (PET) imaging

## Abstract

**Rational:**

Deep learning (DL) has demonstrated a remarkable performance in diagnostic imaging for various diseases and modalities and therefore has a high potential to be used as a clinical tool. However, current practice shows low deployment of these algorithms in clinical practice, because DL algorithms lack transparency and trust due to their underlying black-box mechanism. For successful employment, explainable artificial intelligence (XAI) could be introduced to close the gap between the medical professionals and the DL algorithms. In this literature review, XAI methods available for magnetic resonance (MR), computed tomography (CT), and positron emission tomography (PET) imaging are discussed and future suggestions are made.

**Methods:**

PubMed, Embase.com and Clarivate Analytics/Web of Science Core Collection were screened. Articles were considered eligible for inclusion if XAI was used (and well described) to describe the behavior of a DL model used in MR, CT and PET imaging.

**Results:**

A total of 75 articles were included of which 54 and 17 articles described *post* and *ad hoc* XAI methods, respectively, and 4 articles described both XAI methods. Major variations in performance is seen between the methods. Overall, *post hoc* XAI lacks the ability to provide class-discriminative and target-specific explanation. *Ad hoc* XAI seems to tackle this because of its intrinsic ability to explain. However, quality control of the XAI methods is rarely applied and therefore systematic comparison between the methods is difficult.

**Conclusion:**

There is currently no clear consensus on how XAI should be deployed in order to close the gap between medical professionals and DL algorithms for clinical implementation. We advocate for systematic technical and clinical quality assessment of XAI methods. Also, to ensure end-to-end unbiased and safe integration of XAI in clinical workflow, (anatomical) data minimization and quality control methods should be included.

## 1. Introduction

**Figure 1:**
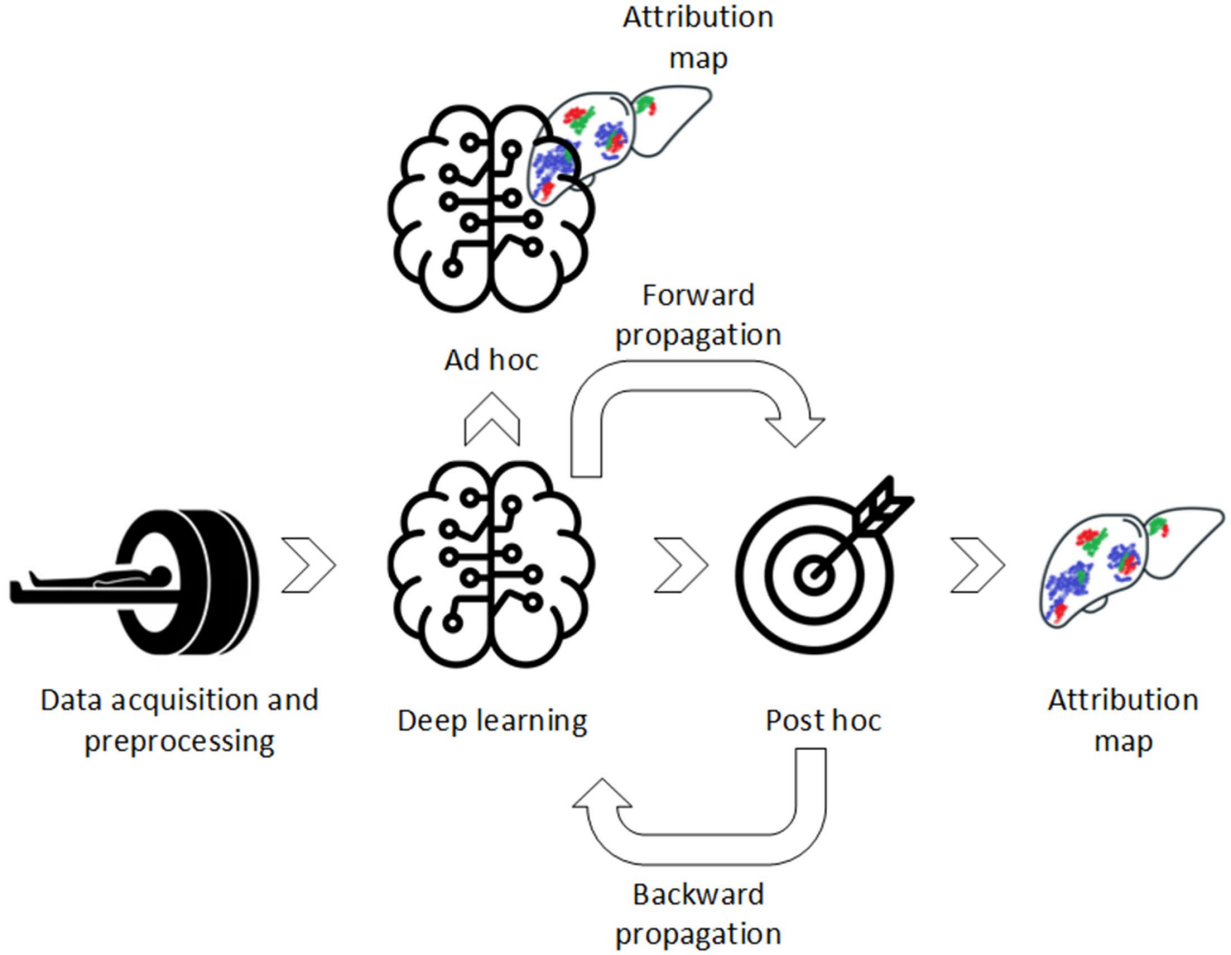
Conceptual difference between *post hoc* and *ad hoc* XAI methods.

Computer-aided diagnostics (CAD) using deep learning (DL) have been widely used in diagnostic imaging for various diseases and modalities (1–5). It shows almost similar or superior performance in comparison to medical professional aided diagnostics and therefore has great potential to be introduced in clinical workflow (6). However, despite the promising results, DL algorithms have not achieved high deployment in clinical practice yet. Unlike simpler machine learning (ML) approaches, DL algorithms do not require manual extraction of features depending on volumes of interest (VOIs) annotation. Instead, DL algorithms extract features in an unsupervised way, i.e., extract features without *a priori* defined assumptions and regulations. Ideally, efficient learning and explainability, i.e., understanding of the underlying DL model, should work together in synergy (Figure 1). Although DL algorithms have superior learning capabilities, they lack transparency due to this underlying black-box mechanism. Therefore, the DL algorithms are difficult to validate, i.e., which features trigger model decision, and lack trustworthiness which is one of the main causes of its low deployment (7–9).

To close this gap, transparency of these DL algorithms should be improved to provide the medical professional and other stakeholders with a pragmatic explanation of the model its decision (10). Explainable artificial intelligence (XAI) can mitigate this gap, because their attribution (i.e., feature importance) methods provide the user with information on why a specific decision is made. This way the user can back propagate the models decision to target specific attributions present in the image. XAI may, therefore, have the potential to be used as a new imaging biomarker (IB) in routine management of patients. In other words, XAI may be able to function as an indicator of normal and/or pathogenic biological processes, which can complement medical professionals in medical decision-making. Also, XAI may provide new insight in disease characteristics, which alternatively can be used as an indicator of responses to an exposure or (therapeutic) intervention. However, XAI should also provide transparency about the quality/legibility of its decision, explanation, and (possible) associated errors. So, before XAI can be used as an useful and trustworthy IB for either testing research hypotheses, or clinical decision-making, it must cross “translational gaps,” through performing and reporting technical validation, clinical validation and assessment of cost-effectiveness (11, 12). Also, the new European Medical Device Regulation (EU MDR) endorses strict regulations regarding transparency that need to be met before such a tool can be implemented in clinical practice (13). XAI may be one of the keys to more transparent, ethical (unbiased) safe and trustworthy deployment of DL algorithms in clinical practice, but better understanding of current practice is required.

This literature review addresses the XAI methods related to DL algorithms in medical imaging. We limit the scope of this review to (functional) magnetic resonance (MR), computed tomography (CT), and positron emission tomography (PET) imaging, which are three of the major cross-sectional imaging modalities. Also, we will try to establish a definition of what high quality explanation means at the end of this review.

## 2. Materials and methods

A systematic search was performed in the databases: PubMed, Embase.com and Clarivate Analytics/Web of Science Core Collection. The timeframe within the databases was from inception to 3rd October 2022 and conducted by GLB and BdV. The search included keywords and free text terms for (synonyms of) “explainable” or “interpretable” combined with (synonyms of) “artificial intelligence” combined with (synonyms of) “medical imaging.” A full overview of the search terms per database can be found in Supplementary Tables 2–4. No limitations on date or language were applied in the search.

To be included in this literature review, studies had to meet the eligibility criteria presented in Table 1. Included studies were classified based on *post* and/or *ad hoc* analysis (Figure 1):

- *Post hoc* methods: These refer to XAI methods that are used after DL model development;
- *Ad hoc* methods: These refer to XAI methods that are used during DL model development.

**Table 1:** Eligibility criteria for inclusion/exclusion.

Eligibility criteria	
Inclusion criteria	Exclusion criteria
XAI used and well describe in the method and result section	Either XAI is not used or is not well described in the method and/or result section
Medical image data available and used as input for DL model MRI; CT; PET	Either no medical image data available or not used as input for DL model Either not MRI; Or CT; Or PET
DL model used	No DL model used

Additional background literature was included to provide (in-depth) information of the XAI methods. This was done through a specific search in PubMed.

In the result section, a general taxonomy of the attribution methods will be provided. Subsequently, per XAI method a (technical) conceptual explanation, its application, its advantages/disadvantages and a comparison with other XAI methods will be provided. Also, we will address the translation gaps present in the literature and a flowchart to *a priori* determine which XAI method to use in medical imaging will be provided. The structure of the flowchart is based on the taxonomy of the available XAI methods as present in the result section of this manuscript and based on from our perspective XAI important disease characteristics identified from the included literature. In addition, we will discuss metrics used in literature for technical and clinical quality assessment of these XAI models. Finally, the current and future direction in this field will be summarized. In Supplementary material a more extensive technical explanation is provided per XAI method.

## 3. Results

**Figure 2:**
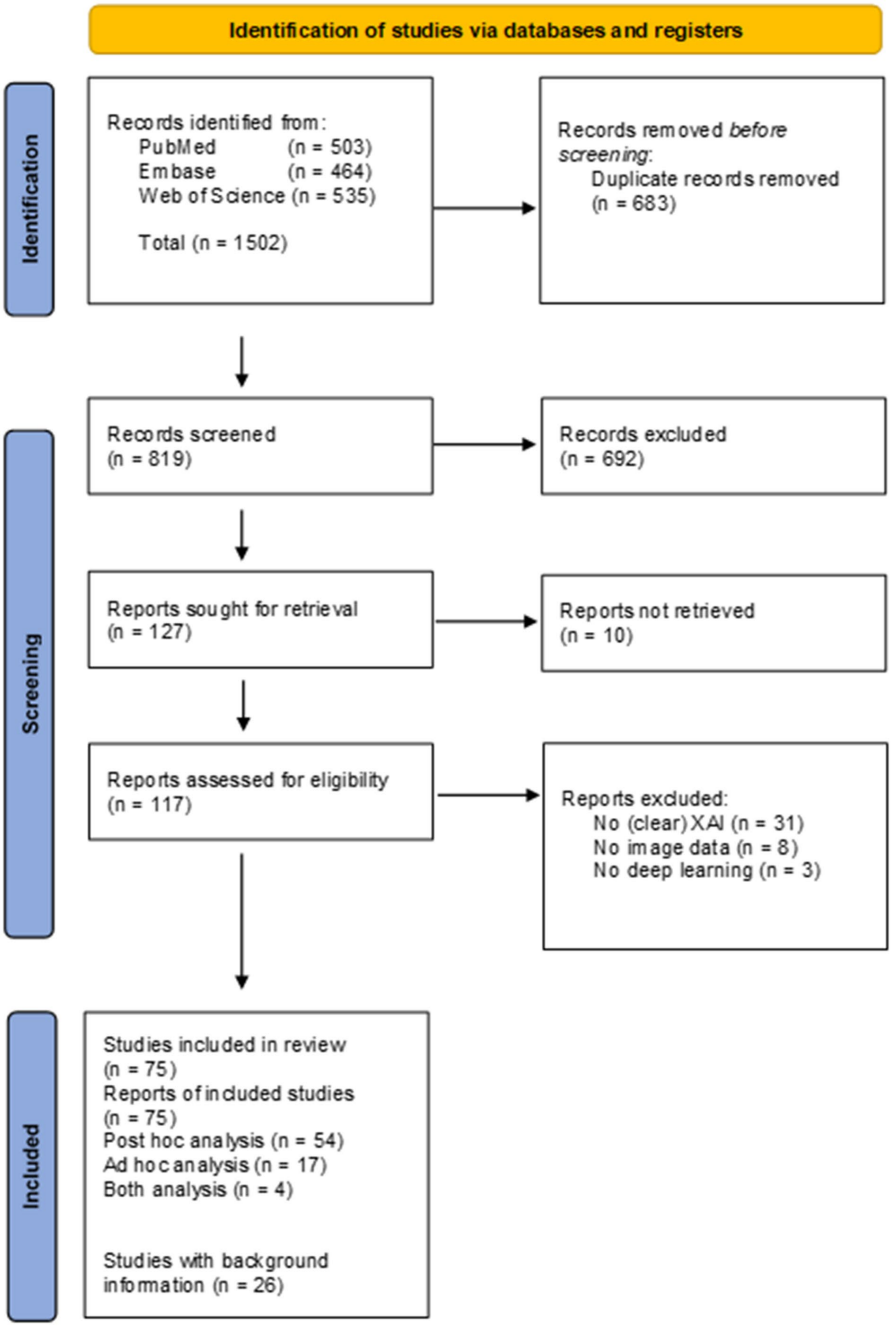
PRISMA flow-chart.

Searches of the literature databases resulted in the inclusion of a total of 117 studies (Figure 2). From the 117 studies, 10 did not have full-text available, 31 did not use or did not clearly describe the usage of XAI methods, eight did not use (medical) image data and three did not use DL, and therefore these were excluded from the review. Of the 75 studies included in the review, 54 studies reported data from *post hoc* analysis, 17 reported data from *ad hoc* analysis and four reported data from both *ad hoc* and *post hoc* analysis. A total of 24 additional studies were included to provide background information. Supplementary Table 1 presents an overview of the 75 studies included in the review.

### 3.1. Taxonomy of XAI methods

**Figure 3:**
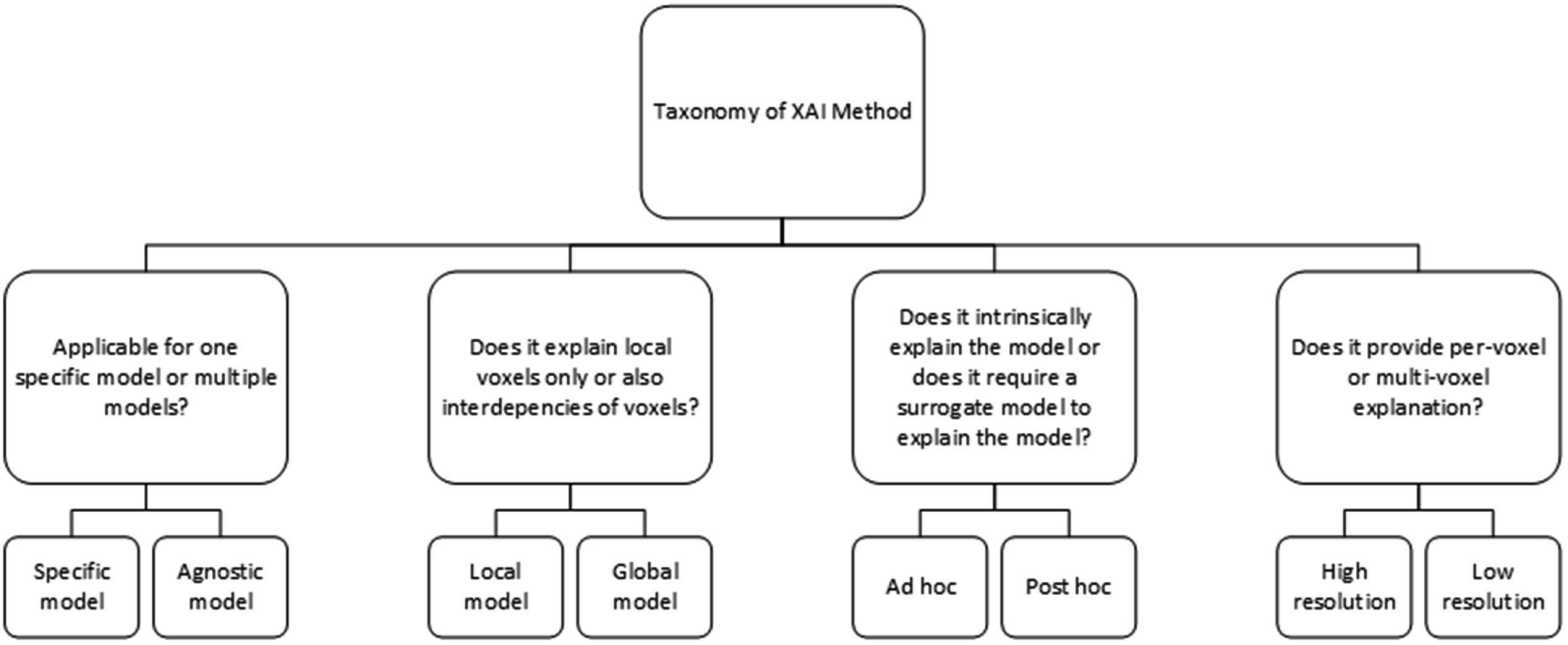
Taxonomy of XAI methods.

The XAI methods in this study are classified based on the XAI taxonomy as shown in Figure 3. *Post hoc* analysis provides model explanation after the classification is made, i.e., an AI model that is able to learn, but requires an additional model to provide an explanation. On the contrary, *ad hoc* explanation models are AI models, which are designed to be intrinsically explainable, i.e., a model that is both able to learn and to explain. Agnostic models are XAI methods that are able to explain multiple (technical) different AI models, while other XAI methods only work with one specific AI model such as a convolutional neural network (CNN). Global XAI methods are models, which are able to capture per-voxel attribution and inter-voxel dependencies, while local XAI methods are only able to provide per-voxel attribution. High-resolution XAI provides a per-voxel attribution value, while low resolution XAI provides a single attribution value for multiple voxels.

### 3.2. *Post hoc* XAI methods

The majority (~75%) of the DL algorithms in this literature study used *post hoc* XAI methods due to its wide availability and its plug-and-play deployment. In the following section, the *post hoc* methods will be divided into gradient-propagation methods, perturbation methods and briefly segmentation and radiomic methods will be discussed. An overview of the *post hoc* attribution methods are shown in Table 2 (and a more extensive explanation in Supplementary material: Appendix A).

**Table 2:** The different *post hoc* XAI methods scored [low/no (red), average (orange), and high/yes (green) performance] based on target specificity, spatial-resolution and local/global voxel dependency capability, model agnostic, and technical simplicity, respectively.

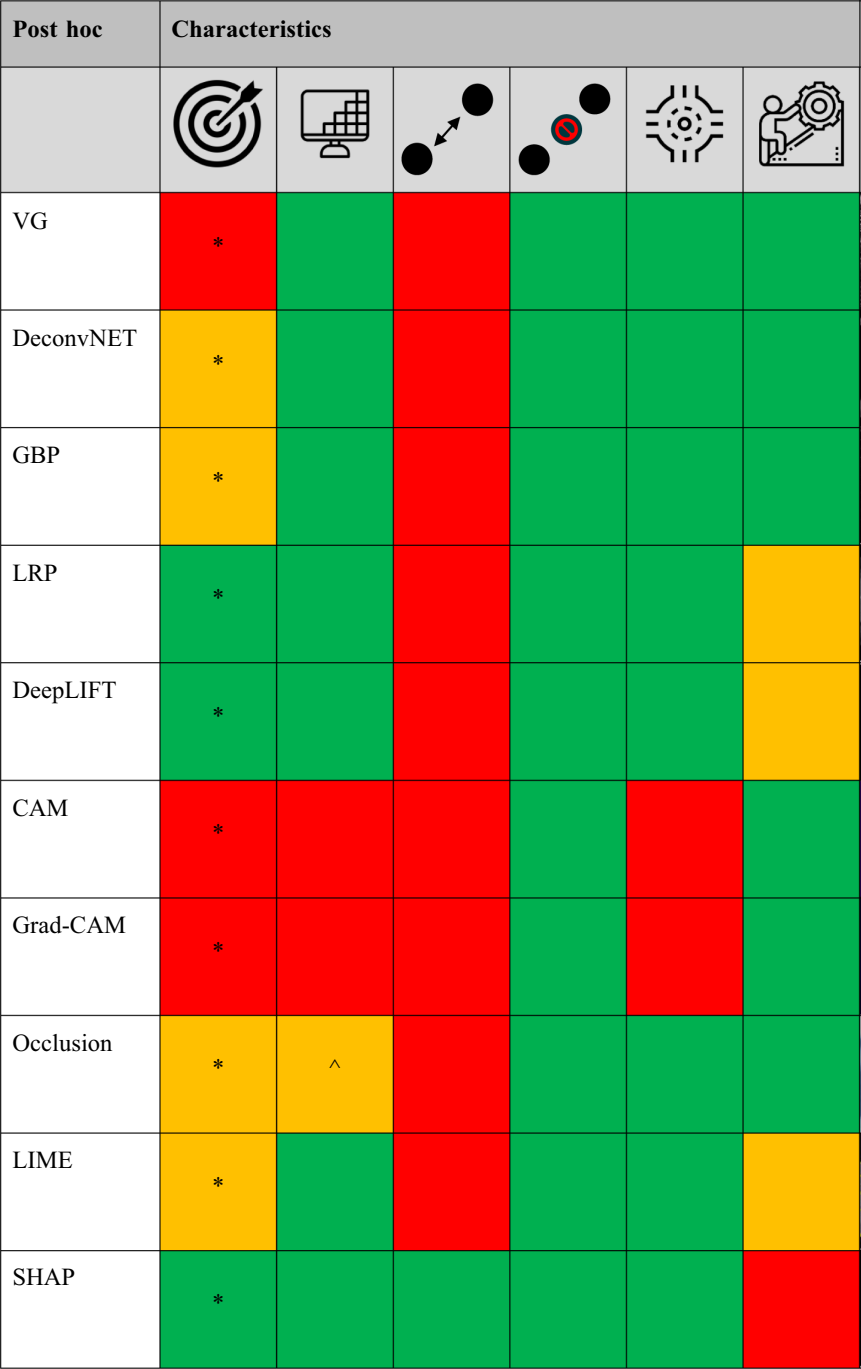

*Depends on DL model convergence. ^Depends on occlusion method.

#### 3.2.1. Gradient-propagation approaches

##### 3.2.1.1. Vanilla gradient (VG)

VG is a XAI method that create an attribution map by calculating gradients over the layers using a single forward and backward propagation, i.e., the input image is fed into the AI model and an output score is calculated (forward) and subsequently the dependence (gradient) between the neurons/convolution layers (subunit of the AI network that learns/extracts features from the input image) and the output is calculated (backward) to create an attribution map. Due to its simplicity, it is an intuitive attribution method and it requires low computational power. An attribution based framework called NeuroXAI compared VG and other attribution based visualization methods for MRI analysis of brain tumors (14). These methods were both utilized for classification and segmentation feature visualization. In comparison to the other attribution methods, VG generated noisy attribution maps and suffers from gradient saturation, i.e., change in a neuron does not affect the output of the network and therefore cannot be measured. In a different study in which the contrast enhancement phase from CT images is predicted, similar results were seen using VG for feature visualization (15). In addition, VG lacks the ability to differentiate between classes (e.g., healthy vs. disease) (16). This illustrates that VG lacks ability to generate clear and class discriminative attribution maps.

##### 3.2.1.2. DeconvNET

DeconvNET is effectively an equivalent of VG apart from the way it calculates the gradient over a Rectified Linear Unit (ReLU) function (17), i.e., a linear function that will output only positive input values and helps with improving model convergence during model training. TorchEsegeta, a framework for interpretable and explainable image-based DL algorithms, compared multiple attribution methods for interlayer CNN visualization in the segmentation of blood vessels in the human brain (18). VG and deconvNET provided more human-interpretable results than the other attribution methods (e.g., DeepLIFT and GradCAM++), since they mainly focused on the vessels, while other methods also showed non-vessel activation.

##### 3.2.1.3. Guided back propagation (GBP)

GBP both incorporates the VG and the deconvNET (19). This results in fewer activated voxels and therefore in less noisy attribution maps than by using each method individually. In the NeuroXAI framework, GBP showed target specific attribution maps with indeed less noise in comparison to VG (14). In a study performed for predicting brain abnormalities using MRI, an additional smoothing function to the GBP was proposed to suppress the amount of noise and the effect of non-target specific attributions even more (20). The attribution maps showed low noise and accurate localization of a range of morphological distinct abnormalities. However, although GBP may show less noisy attribution maps, it may also result in overly sparse attribution maps, which are not useful for complete image characterization (21).

All three gradient based methods are very sensitive to understand how the neural network layers extract features, but are not class discriminative. Also, because of ReLU and pooling layers, local gradients may saturate. Therefore, important features may vanish over the layers in the network and that may result in incomplete model explanation or even focus on irrelevant features.

##### 3.2.1.4. Layer-wise relevance propagation (LRP)

LRP is a XAI method that operates by propagating the class score backward over the neural layers to the input image using LRP specific rules (22). The concept of LRP is to conserve inter-neuron dependency, i.e., what has been received by a neuron layer will be redistributed to the following lower layer in equal quantity. The decomposition is based on propagating relevance scores between the neurons instead of gradients and therefore, we tackle the difficulties of the saturation problem. In a study for screening of abdominal aortic aneurysm in CT images (23), LRP showed clear class difference based on activation difference in the lumen of the aorta. However, high activation for both classes was also seen in the vertebra, which indicates that either the DL model is biased, the DL model did not converge, the vertebra is a confounder, or that LRP also incorporates non-target specific features in its attribution map. A similar result was seen for COVID-19 classification, in which LRP was not able to visualize target-specific features (24). However, other studies showed class-discriminative regions and precise localization of lesions using LRP (25, 26). This difference may be explained by differences in DL model performance, biased data and LRP configuration, although there may not be one absolute reason.

##### 3.2.1.5. DeepLIFT

DeepLIFT is a XAI method that uses a neutral reference activation (e.g., neuron activation of CT scan without pathology/disease) to solve the saturation problem (27). This reference activation is used to describe the change of a new neuron activation in comparison to the reference activation. From these differences, contribution scores are calculated for each neuron to compute an attribution map. DeepLIFT was compared with LRP and VG for identification of Multiple Sclerosis (MS) patients on MRI (26). This was done by perturbation of the three attribution maps for three VOIs. From quantitative assessment, it can be seen that DeepLIFT performs slightly better than LRP and much better than VG in extracting target-specific features. Both LRP and DeepLIFT are able to tackle gradient saturation, which may be the reason why it performs better than VG in this classification task.

##### 3.2.1.6. Class activation map (CAM)

CAM is one of the most well-known model specific attribution methods (28, 29). It uses a Global Average Pooling (GAP) layer instead of multiple dense layers, which introduces linearity after the last convolution layer and before the final dense layer. Since CAM only uses features from the last convolution layer, low-dimension attribution maps are generated. Therefore, the low-dimension CAM is able to visualize whether a model is able to roughly focus on specific targets, but due to its low specificity, it lacks discriminative power to accurately characterize class based features (30, 31). Perturbation analysis of multiple attribution methods also showed that gradient based methods have higher specificity than CAM (15). Yet, CAM can be discriminative in classification tasks in which the classes have clear visual differences, e.g., healthy brain vs. Alzheimer’s brain (32) or by performing patch based (more focused) tumor analysis instead of whole image tumor analysis (33, 34).

##### 3.2.1.7. Gradient-CAM (Grad-CAM)

Employment of XAI methods has showed tremendous growth due to COVID-19 detection (35). In general you can distinguish these methods based on using the whole CT image, or only using a segmentation of the lungs for COVID-19 detection. Especially, whole image based COVID-19 detection showed major performance difference in attribution mapping. Grad-CAM, an extension of CAM, was the most used attribution method and showed both very specific (36, 37) as also non-specific attributions (24, 38–41), but was overall able to roughly locate the potential COVID-19 lesions to make accurate predictions. To remove the influence of non-target specific features, *a priori* segmentations of the lungs was proposed (42–47). This way both the DL algorithms as the XAI methods can only extract features from the lungs. This anatomical based XAI method showed higher specificity than by using the whole CT image using Grad-CAM. This shows that DL and XAI methods benefit from medical based data minimization, in other words reducing the amount of trainable features and/or removing non-informative features from the input image.

Similar non-target specific attribution maps were also seen for the automated grading of enlarged perivascular spaces in acute stroke (48) and cerebral hemorrhage detection (49) using the whole image (without data minimization). Similar as for the COVID-19 studies to solve this specificity problem, *a priori* anatomical segmentation was used to classify and visualize mortality risks based on myocardial PET (50), Alzheimer’s disease (51) and schizophrenia based on MRI (52). However, although data manipulation suppresses the presence of non-target specific features, Grad-CAM still suffers from low specificity due to its low-dimensional attribution maps (43, 53). In a study for classification of lung cancer histology based on CT images, the authors suggested that based on the Grad-CAM attribution maps, the activated features around the tumor correspond to regions harboring occult microscopic disease (2). However, this is more likely caused by this low-dimensionality characteristic of Grad-CAM, because CT does not have high enough spatial resolution to detect these microscopic diseases.

Similar to CAM, Grad-CAM can be class discriminative in case of classification tasks with clear radiological difference between the classes (5, 54–58). However, in case of tasks with less obvious radiological differences, e.g., predicting survival based on tumor characteristic, Grad-CAM lacks fine-grained details, complementary attribution methods should be used such as VG and GBP (15, 18). A study that combined GBP with Grad-CAM, a method called guided Grad-CAM (gGrad-CAM), showed better localized attribution maps with higher resolution in MRI analysis of brain tumors (14). This advocates for combining the advantages of attribution methods for human-interpretable and precise model visualization.

There have been multiple other improved variation of Grad-CAM, such as Grad-CAM++. Grad-CAM++ has been introduced to provide better localization of target-specific features than Grad-CAM (59). Grad-CAM averages the gradients of the feature maps, which may suppress the difference in importance between the different gradients. Grad-CAM++ replaces this with a weighted average, which measures the importance of every unit of a feature map. It showed more target-specific attribution maps than Grad-CAM in the prediction of knee osteoarthritis using MRI (25).

The advantage of gGrad-CAM is clearly shown in a study where they compared different attribution methods for brain glioma classification (14). Grad-CAM provided the least noisy attribution maps and GBP provided attribution maps with high resolution but not class-discriminative. However, gGrad-CAM provided both class-discriminative as high resolution maps in which the edges of the tumor are highlighted instead of the whole tumor. Similar results were also seen for classification of frontotemporal dementia (60), although the skull was seen important for the classification as well.

However, non-target specific features in attribution maps do not only arise because of underperformance in DL algorithms and/or attribution methods. Artifacts can also play a major role in tricking DL algorithms and attribution methods (61). That is why it is important to have high quality data, perform (medical based) data minimization and have *a priori* (DL-based) quality control methods to detect bias present in the data (62, 63). In addition, it is also not always trivial what convolution layer should be used to compute the attribution map (64). Deeper layers may have higher hierarchical structures, but may suffer from low specificity and therefore using a shallower layer may contain more informative features.

#### 3.2.2. Perturbation XAI methods

##### 3.2.2.1. Occlusion mapping

Occlusion mapping is a simple to perform approach that reveals the feature importance of a model using systematic perturbation/conditioning over the image (e.g., replacing input pixels with zeros). In contrast to previous methods, occlusion maps do not take the feature maps into account, but only the different patches (grid- or atlas-wise combination of multiple pixels) of the input image. Therefore, it is a very intuitive method, which can easily be adapted to specific occlusion analysis. An example of this is a study that investigated the use of DL algorithms in predicting and visualizing Alzheimer’s disease and Autism using MRI. But instead of rectangles, the Harvard-Oxford cortical and subcortical structural atlas was used for occlusion mapping (65). This provides a method that can easily be compared with more traditional atlas based analysis and therefore provides a medical based, transparent and intuitive visualization of the DL algorithm.

Randomized Input Sampling for Explanation (RISE) is an equivalent of occlusion mapping, but instead of systematic perturbation of the input image, it generates multiple random perturbation maps, which are pointwise multiplied with the input image (66). Another occlusion method is square grid, where perturbation is performed using square grid divisions (62). These methods produce intuitive results, but are too rigid to follow anatomical/pathological structures present in the images, and require large computational power due to many forward and backward propagations.

##### 3.2.2.2. Local interpretable model-agnostic explanations (LIME)

Instead of a predefined or random occlusion function, LIME perturbates super-pixels, which are a group of pixels that share common pixel/voxel characteristics. For COVID-19 detection using CT, super-pixels followed anatomical/pathological structures/characteristic of the image and therefore gave a better representation of the image than the previous occlusion methods (67–69). However, since LIME uses super-pixels as a whole, it provides occlusion maps with relatively low specificity. Also, from these COVID-19 studies it can be seen that non-target specific features (e.g., chest wall) show high activation. This suggests that also occlusion mapping suffers from non-target specific activation. In addition, LIME requires initialization parameters (kernel size, maximum distance, etc.) to compute super-pixels, which can be difficult to optimize.

##### 3.2.2.3. SHapley additive exPlanations (SHAP)

SHAP is an advanced XAI algorithm that calculates SHAP values, which represent the attribution of each voxel to the change of the expected model prediction when conditioning on that voxel using reference samples (70). DeepSHAP is an extension of SHAP and works in an almost similar way as DeepLIFT. It can provide both local as global explanation based on individual pixels/voxels, but also whether a pixel/voxel is negatively associated or positively associated with the predictive class. Because of this, DeepSHAP may be difficult to interpreted as is shown in a study to predict brain tumors using MRI (67). However, in a study in which the volumetric breast density on MRI was calculated using DeepSHAP, intuitive DeepSHAP maps were created (71). This difference may be the result of difference in data size and quality between the studies, but may also be impacted by the quality of the reference samples to create the attribution maps. Also, because of the required reference samples, DeepSHAP may not work optimal in classification tasks where there are substantial (non-)rigid anatomical/pathological variation present in the images. Feature explanation may therefore be negatively impacted by anatomical differences between the reference samples and the input image and therefore may show non-specific attributions.

#### 3.2.3. Probability maps, deep feature maps, radiomics, and physics/clinical data

Previous described *post hoc* attribution methods predominantly focus on classification models, which are trained using weak labels, i.e., one label for the whole image. In contrast, segmentation DL algorithms use voxel-level annotations and compute voxel-level probability maps. Therefore, these probability maps are less complex to understand.

These probability maps were used to detect prostate lesion from multi-parametric MR sequences, which were easily interpretable and it allowed to perform prostate lesion analysis in new image data (72, 73). Similar probability maps were also created to detect lumbar spine MR intensity changes (74). However, further specific Modic type categorization was performed using a non DL-based, but interpretable signal-intensity based nearest neighbor algorithm.

These segmentations can also be used to explore radiomic (e.g., intensity, morphology, and texture) based differences between classes. A joint detection and radiomic based classification algorithm was developed to explore the radiological difference between COVID-19 and community acquired pneumonia and showed clear difference between the two classes using understandable radiomic features (75). A similar approach was used for detection and classification of lung nodule malignancies (76, 77).

Although these methods (partly) tackle the problem of black-boxes, voxel-level annotation is very cumbersome and radiomic analysis depends on accurate VOI annotations, and *a priori* defined assumptions and regulations. This may suppress the full potential of DL algorithms and therefore have a possibility to underperform.

Another explainable method, is the use of deep feature maps (intermediate attribution maps) of the DL-based models (78). These deep feature maps provide the user with attributions maps of the intermediate model layers, which visualizes the underlying feature extraction mechanism used by the DL-based model. It therefore can give the user an understanding of what features are used, but more importantly how these features are processed throughout the model.

Physics-based AI models could also aid in higher transparency, as these models can explain feature extraction through well-defined mathematical formulas/assumptions, i.e., physics-aware AI. These models incorporate physics/mathematical knowledge prior to training. However, this approach is predominately used for image reconstruction and has low application/added-value for classification (yet) (79).

Also, clinical data (e.g., patient history) could aid in better performance and transparency of The AI algorithms. for detection of prostate cancer using MRI, clinical data improved The diagnostic performance significantly (73). In a different study, both clinical data and radiomics features showed a complementary role in the prediction of EGFR and PD-L1 status using CT images (30).

### 3.3. *Ad hoc* XAI models

*Ad hoc* XAI models are intrinsically able to learn and explain, which is different to the DL models that predominantly focusses on learning to achieve high performance (learning) and require a *post hoc* XAI algorithm to explain model behavior. An overview of the *ad hoc* attribution methods are shown in Table 3 (and a more extensive explanation in Supplementary material: Appendix A).

**Table 3:** The different *ad hoc* XAI methods scored [low/no (red), average (orange), and high/yes (green) performance] based on target specificity, spatial-resolution and local/global voxel dependency capability, model agnostic, and technical simplicity, respectively.

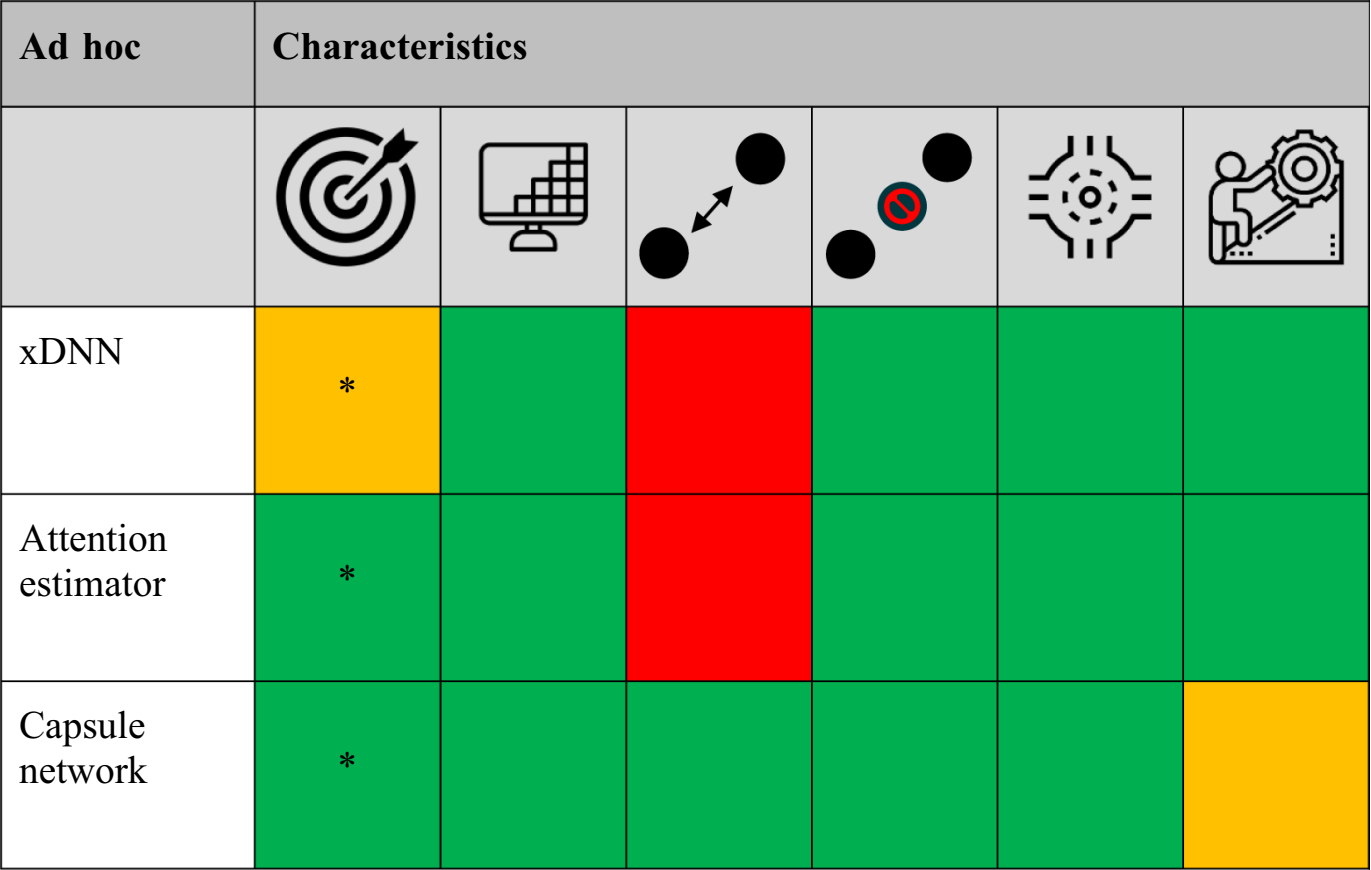

*Depends on DL model convergence.

#### 3.3.1. Explainable deep neural network (xDNN)

xDNN is a XAI method that uses a prototype identification layer in the network to identify new data samples based on similarity to predefined data samples (prototypes) (80). For this, representative prototypes need to be selected for each class, which can be a difficult task, especially in case of a cohort with a wide variety in disease morphology. Also, difference in class morphology is not always trivial and therefore obtaining representative prototypes can be difficult. However, xDNN can be very powerful in tasks where there is known difference between classes, as is the case for COVID-19 screening (81–84) and artifact detection (63). In these studies representative prototypes were used to assess new images based on their similarity. This provides the user with transparent and intuitive model explanation, which in some way mimics the way we humans extract features based on previous experience.

#### 3.3.2. Capsule networks

Capsule networks are described to be the new sensation in DL, since they are able to eliminate the pose and deformation challenges faced by CNNs, require less data and less computational power (85). A capsule tries to describe the presence and the instantiation parameters (orientation, thickness, skewed, position, etc.) of a particular object (e.g., tumor or lung) at a given location as a vector. Subsequently, the vectors from a lower capsule layer try to predict the output for the higher layer based on the instantiation parameters. Lower layer vectors with high agreement are routed to the following layer and the other vectors are suppressed, ideally resulting in only target specific attribution maps. A study proposed a novel capsule network-based mixture of expert (MIXCAPS) for detection and visualization of lung cancer nodules in CT images (86). MIXCAPS is an extension of the traditional capsule network, where instead of a single CNN, a mixture of (expert) CNNs specialized on a subset of the data and an ensemble of capsule networks is used. The authors compared MIXCAPS with a single capsule network, a single CNN and a mixture of CNNs and showed superior performance using MIXCAPS. However, its full potential has not been shown yet and requires further understanding before it will be used as the standard DL algorithm in this field.

#### 3.3.3. Attention mapping

A trainable spatial self-attention mechanism is in contrast to *post hoc* attention mechanisms, trained during model training to support (important) feature extraction (87) and replaces traditional non-learnable pooling operations (e.g., max-pooling). Spatial attention mapping uses attention estimators to compute attention mask from a convolution layer as a goal to extract important local feature vectors. Attention mapping showed high correspondence between attention scores of specific regions and classification score in a study that assessed interpretable imaging biomarkers for Alzheimer’s disease using MRI (88). In addition, attention mapping for COVID-19 detection showed better capabilities to extract more complex and scattered regions (24, 89). Attention mapping has also showed superior target-specific feature extraction in inverted papilloma and nasal polyp classification using CT (90), adenocarcinoma screening using CT (91) and segmentation of multiple organs from MRI (92).

Attention mapping has also been investigated in combination with Multi Instance Learning (MIL). MIL tries to tackle the downsides of weak labels and labor intensive per-voxel annotation. Instead MIL uses a set of labeled bags, each consisting of multiple instances (slices). In case of binary classification, a bag will be annotated negative if all the instances in the bag are negative (e.g., no presence of disease) and will be positive if there is at least one instance in the bag which is positive. Therefore, MIL intrinsically provides a more interpretable decision and in combination with attention mapping it gives insight into every voxel its contribution to the bag label. This combination have been used for the detection of COVID-19 using CT and showed more precise and complete detection of the infection areas of COVID-19 than weak labeled methods (93, 94). A similar method has been used to predict EGFR mutation status using CT and improved the interpretability of the model (95). This indicates that attention mechanisms (in combination with MIL) provide more spatial resilient CNNs, as it mimics the human behavior of focusing on more relevant features, while suppressing irrelevant features.

An alternative attention mechanism has been suggested for detection of COVID-19 from CT by feature encoding using a gated recurrent neural network in the horizontal and vertical direction using a feature block grid (96). In contrast to traditional CNNs, this mechanism allows to capture the horizontal and vertical dependencies of the features present in the image. This attention mechanism helps to make the model interpretable. However, it lacks specificity due to its grid-wise attention mechanism.

### 3.4. Explainability quality of attribution methods

Performance assessment of DL algorithms is almost always expressed in terms of diagnostic performance (e.g., accuracy, sensitivity) or overlap (e.g., Dice coefficient) with the gold standard. Although CNNs are seen as the current state-of-the-art algorithms in this field, there is no clear consensus what XAI method has superior performance over the other methods. One of the problems with these XAI methods is that the performance of the attribution methods is often not expressed in measurable (quantitative) metrics. Most comparisons are performed solely on visual inspection, which is susceptible to human subjectivity, especially in case of non-trained readers. Current literature therefore lacks high-quality and objective technical and clinical assessment of the attribution methods, which makes objective comparison between the XAI methods difficult.

However, from the studies that used technical and clinical quality assessment, in general you can divide measurable metrics into human-based and computer-based derived metrics. Human-based metrics predominantly use correspondence scores to assess overlap between decision relevant VOIs and the gold standard VOIs. In a study where they assessed the correspondence of the attribution map with the aorta, the radiologist used a 5-point Likert scale to determine correspondence (23). An equivalent score, the mean alignment index (MAI) was used to evaluate the attribution map for COVID-19 detection (44). Another study measured the effect of diagnostic performance with and without attribution maps (spinal Modic maps) (74). First, they provided three radiologist with a MRI without the attribution maps and after a 4-week washout period, the radiologist regraded the same dataset with the attribution maps. Although a 4-week washout period might not be sufficient, such methods are able to validate the effect of attribution methods in complementing medical professionals in medical decision making and therefore helps improving the trustworthiness of these algorithms in this field.

Computer-based metrics also use metrics to measure overlap between the attribution maps and a gold standard. A study calculated correspondence between the attribution maps with brain tumor segmentations using a localization hit and the intersection over Union metric (55). In other studies, correlation analysis was performed to compare pneumonia ratio between radiologists and thresholded attribution maps for COVID-19 detection (24) and between attribution scores of brain regions and classification accuracy in Alzheimer’s disease (88). Another method proposed is the use of perturbation of the input image based on the attribution maps (26). The idea behind this is that important features from the attribution map should correspond with important features from the input image, which is expressed as the area over the perturbation curve (AOPC). So the more the prediction score decreases by perturbation, the better an attribution method is capable to identify relevant input features, resulting in a high AOPC.

### 3.5. Disease specific XAI

**Figure 4:**
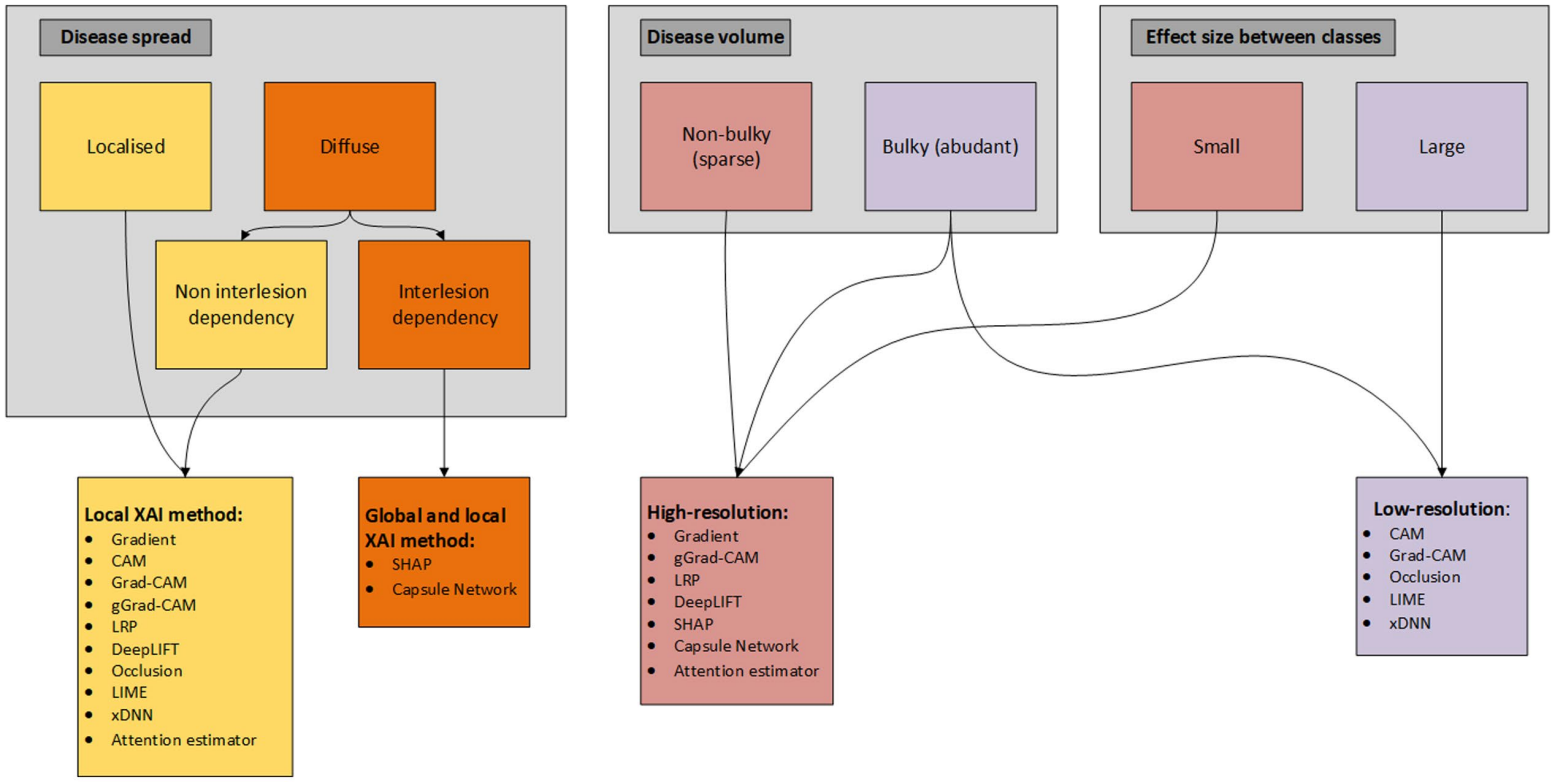
Flowchart of XAI methods applicable for disease specific characteristics.

Utilization of disease-specific XAI is not unambiguous and therefore we propose a flowchart (based on taxonomy of the XAI methods) to determine what XAI methods present in the literature are from our perspective (most) applicable based on disease specific characteristics/patterns (Figure 4). In this flowchart we differentiate between local and global and low- and high-resolution XAI methods, what we think are two (important) taxonomies that can be determined *a priori* for the development of XAI methodology. Differentiation of the XAI methods is based on disease spread, disease volume and effect size between the classes. Disease spread is divided into localized (e.g., only primary tumor) and diffuse (e.g., diffuse large B-cell non-Hodgkin lymphoma), where diffuse spread is again subdivided into non-interlesion (e.g., predicting non-Hodgkin vs. Hodgkin lymphoma) and interlesion (e.g., prediction of overall survival for Hodgkin lymphoma) dependency. Although the difference between the two seems small, a non-interlesion dependency can be described in terms of a regional (small ROI/VOI) linear relation with the output [e.g., (non-)presence of bone metastasis in Hodgkin vs. non-Hodgkin patients], while interlesion interaction requires an explanation/relation for all pixels/voxels (e.g., relationship between primary tumor, lymph node and distant metastases). Interlesion (voxel) interaction therefore requires both local as global XAI, while localized disease only requires a XAI method to extract local features. Disease volume is divided into non-bulky (e.g., stage I pancreatic cancer) and bulky (e.g., diffuse large B-cell non-Hodgkin lymphoma). The effect size, i.e., the magnitude of the difference between classes, may in some cases be more difficult to determine *a priori*. Yet, we divide the effect size in small (e.g., predicting progression free survival in stage III colon cancer) and large (e.g., predict presence of glioma in brain vs. healthy brain). This flowchart can be helpful for researchers to determine *a priori* what XAI methods currently present in literature can aid in explaining their DL model. However, in the end researchers should determine how the complexity of the AI task compares with the complexity of the XAI method and therefore the flowchart should only be seen as an additional tool for XAI application.

## 4. Discussion

There has been growing interest in the deployment of XAI to explain DL black-boxes in the field of MR, CT, and PET imaging. However, this review demonstrates that there is a variety of XAI methods available and that there is currently no clear consensus present in literature on how and what XAI should be deployed to realize utilization of DL algorithms in clinical practice. Although a variety of XAI methods are proposed in literature, technical and clinical quality assessment of these methods is rarely performed. Also, there is little evidence of the impact of attribution methods to complement medical professionals in medical decision making and what medical professionals expect and demand of XAI (74). This all illustrates that current XAI methods on their own may not be sufficient to realize deployment in clinical practice, but requires additional/tweaked (XAI) methods to improve transparency and trustworthiness. Therefore, we advocate for an end-to-end solution, which integrates *a priori* data-quality control, data pre-processing, (self-)explainable modules and technical and clinical (X)AI model quality control (26, 74). In addition, to the best of our knowledge we are the first study that provides a guide for current available XAI utilization based on disease/AI task specific characteristics (Figure 4). Also, we have provided a hands-on summary of the (dis-)advantages of each XAI method (Tables 2, 3). Both can be helpful for researchers to *a priori* determine which XAI method can be useful for their disease-specific AI task.

The majority of the studies utilized *post hoc* attribution methods to explain model behavior. For successful employment, these XAI methods should be transparent, explainable and safe for all stakeholders. Current *post hoc* XAI methods are overall able to provide transparent and understandable attribution maps, but show low specificity, resulting in non-target specific attribution maps. Anatomical data minimization seems to suppress the effect of this, but due their intrinsic technical characteristics some still lack to provide class discriminative performance. In recent years, more advanced *post hoc* methods have been proposed, such as DeepSHAP. DeepSHAP uses multiple reference image samples from both classes and is therefore able to provide both positive as negative attributions. Therefore, DeepSHAP enables reasoning both for and against a models decision, which is important to consider for a complete image analysis and diagnosis. Although this provides high model transparency and greater insights, excessive information may result in lower understandability by the medical professional. Also, DeepSHAP may be negatively impacted by anatomical (non-)rigid variation in images and reference images and therefore may not work optimal in medical imaging.

From this perspective it is important to consider what medical professionals consider as complementing information for decision making. It is therefore critical to focus on addressing the epistemic and non-epistemic concerns of this group in specific contexts and occasions of these DL algorithms. These algorithms should be designed in the context of its user, which includes flawless integration in the user’s clinical workflow, respect the autonomy of the user and provide transparent and effective outputs (97). One of the overall issues of XAI is the low specificity of the computed attribution maps, i.e., non-informative attributions make it overly difficult to interpreted the attribution maps. This may be due to the wide non-medical application of these XAI methods, i.e., they are not optimized for medical imaging. Therefore, although these methods may be useful in more simple (non) medical AI tasks, more difficult medical AI tasks may require XAI methods specifically developed for medical imaging. In other words, these systems should be designed around stakeholders/imaging modalities to ensure both transparent and trustworthy outputs.

Although not extensively present in literature, *ad hoc* XAI models do provide intrinsic explanation of their decision and seem to be more target-specific than *post hoc* XAI methods. Self-attention mapping has showed great interest, because it is able to intrinsically explain, showed higher target specificity than *post hoc* algorithms and is also relatively simple to understand and integrate into current systems. However, self-attention mapping is not able to find global feature dependencies, which can be important in disease mapping. Yet, self-attention mapping in combination with DeepSHAP enables it to find global features, which supports to combine *ad hoc* and *post hoc* XAI methods in future research. Another promising XAI method is capsule networks, which are intrinsically able to handle spatial relationship between features and therefore have seen to be more resilient to spatial variance than CNNs. Also, agreement by routing provides an intuitive explanation of which feature belongs to which object. Therefore, capsule networks have been suggested as the new state-of-the-art DL model, but more research is required to explore its full potential.

To ensure unbiased and safe end-to-end integration of DL systems, also data quality control should be performed. Especially for systems with small data exposure, poor data quality can have high impact of the models its reliability. For example measuring the signal-to-noise-ratio for data quality harmonization, DL-based artifact detection model (62, 63) or simple visual inspection can be proposed to provide information of the quality of the data before utilization in the diagnostic DL systems. Also, quality control of the attribution maps should be performed to assess the use of XAI as potential IB. Unfortunately, only few studies (26, 55) implemented quality control systems to assess whether the attribution maps do present target-specific features. The absence of complete and transparent technical and clinical reporting limits the usability of finding in studies and in consequence, the acceptance of XAI as IB in clinical practice. In response to this, a new version of the Transparent Reporting of a multivariable prediction model for Individual Prognosis Or Diagnosis (TRIPOD) Statement was published (12). The TRIPOD Statements provides a checklist for reporting of studies developing, validating, or updating a prediction model for diagnostic and/or prognostic purpose. In combination with standardized modality and/or disease specific implementation guidelines (11), higher transparency and effectivity of XAI as new IB can be utilized in future research.

## 5. Conclusion

High quality explanation is user and task subjective and therefore we require pragmatic explanations to address the concerns of DL algorithms for each stakeholder/imaging modality. *Ad hoc* XAI methods seem to provide state-of-the-art explanation algorithms, which advocates for shifting from *post hoc* to integrating self-explainable modules in the DL models. However, there is (still) no unambiguous (self-)explainable XAI method addressing all concerns, which advocates for combining XAI methods, perform anatomical data minimization and implement data quality systems to ensure end-to-end unbiased and safe system integration into the context of the stakeholder/imaging modality.

Although XAI shows a great potential to be used as IB in clinical practice, technical and clinical quality assessment is currently rarely reported. We recommend the utilization of developing and reporting guidelines, accepted by the AI-community, to ensure a higher transparency and quality of future developed XAI algorithms.

## Author contributions

BV, CM-v, FV, and RB: conceptualization. BV, CM-v, FV, GB, and RB: methodology. BV: investigation, writing—original draft preparation, and visualization. BV, GZ, CM-v, FV, GB, and RB: writing—review and editing. CM-v, FV, and RB: supervision. All authors have read and agreed to the published version of the manuscript.

## Conflict of interest

The authors declare that the research was conducted in the absence of any commercial or financial relationships that could be construed as a potential conflict of interest.

## Publisher’s note

All claims expressed in this article are solely those of the authors and do not necessarily represent those of their affiliated organizations, or those of the publisher, the editors and the reviewers. Any product that may be evaluated in this article, or claim that may be made by its manufacturer, is not guaranteed or endorsed by the publisher.
