# Supplementary material for: Explainable artificial intelligence (XAI) in radiology and nuclear medicine: a literature review
Source: Front Med (Lausanne). 2023 May 12;10:1180773. doi: 10.3389/fmed.2023.1180773 (PMC10213317; doi:10.3389/fmed.2023.1180773)
Supplement: Supplementary file 1 [file Data_Sheet_1.docx]

## **Appendix A: XAI methods explained**

#### Vanilla gradient (VG)

VG calculates the gradient of the cost function corresponding to class $c$ with respect to the input image $f\left( x,y \right)$ [98]. This is achieved by first performing a forward pass of the input image and subsequently the gradients of the class score $S_{c}$ with respect to the input image is computed using backpropagation (Equation 1) to create the attribution map $R_{0}$.

$w=\frac{\partial S_{c}}{\partial f\left( x,y \right)}$ Equation 1

VG is different in comparison to DeconvNET and Guided backpropagation in the way the gradient is calculated over the linear ReLU rectification layer (Equation 2 and Supplementary Figure 1).

$\frac{\partial f\left( x,y \right)}{{\partial X}_{n}}=\frac{\partial f\left( x,y \right)}{{\partial X}_{n+1}}*(X_{n}>0)$ Equation 2

#### DeconvNET

So similar to the VG, backpropagation is performed over the convolution layers to compute the gradient. For the ReLU rectification layer, however, the gradient is computed using the output reconstruction $R_{n+1}$ instead of the layer input $X_{n}$ (Equation 3 and Supplementary Figure 2).

$\frac{\partial f\left( x,y \right)}{{\partial R}_{n}}=\frac{\partial f\left( x,y \right)}{{\partial R}_{n+1}}*(R_{n+1}>0)$ Equation 3

#### Guided backpropagation (GBP)

GBP both incorporates the VG and the deconvNET [19]. This means that for the gradient over the ReLU only positive values of both the output reconstruction $R_{n+1}$ and of the layer input $X_{n}$ are conserved (Equation 4 and Supplementary Figure 3).

$\frac{\partial f\left( x,y \right)}{{\partial R}_{n}}=\frac{\partial f\left( x,y \right)}{{\partial R}_{n+1}}*\left( X_{n}>0 \right)*\left( R_{n+1}>0 \right)$ Equation 4

#### Layer-Wise Relevance Propagation (LRP)

The conservation property in LRP is described by the propagating relevance scores ${{(R}_{n})}_{n}$, where $z_{jn}$ quantifies to which extent neuron $j$ has contributed to neuron $n$ in terms of relevancy (Equation 5 and Supplementary Figure 4).

$R_{j}=\sum_{n} \frac{z_{jn}}{\sum_{j} z_{nj}}R_{n}$ Equation 5

#### DeepLIFT

DeepLIFT uses a neutral reference activation and in this way the change in neuron activation $t$ is described in terms of difference $\Delta f(x,y)$ towards the reference activation $f_{0}(x,y)$. From these differences, DeepLIFT computes contribution scores $C_{\Delta X\Delta f}$ (Equation 6 and Supplementary Figure 5). This way even when the gradient is zero, contribution score can be non-zero, i.e. a local neuron can provide a signal although its local gradient is zero.

$\sum_{i=1}^{n} C_{\Delta X\Delta f}=\Delta f$ Equation 6

#### Class activation map (CAM)

CAM uses a GAP layer instead of multiple dense layers. GAP takes the average of every single feature map $f_{k}(x,y)$ from the convolution layer, resulting in a linear vector. Subsequently, from the weights $w_{c}^{n}$ corresponding to class $c$ for feature map $n$, the CAM is calculated (Equation 7 and Supplementary Figure 6)

${CAM}_{c}\left( x,y \right)= \sum_{k} w_{c}^{n}f_{n}(x,y)$ Equation 7

#### Gradient class activation map (Grad-CAM)

Grad-CAM is based on CAM [99], but in addition it calculates a gradient $\frac{\partial S_{c}}{\partial A_{n}}$ corresponding to the class that flows from the dense layers to the (final) feature maps $k$ using backpropagation. The gradient of the class score is then averaged using GAP ($a_{c}^{n}$) and multiplied with the rectified feature maps $A_{n}$. Subsequently, a ReLU is applied (Equation 8) to generate the Grad-CAM using forward propagation. Using ReLU, only features that have positive influence on the class are allowed.

${GradCAM}_{c}=ReLU(\sum_{k} a_{c}^{n}A_{n})$ Equation 8

#### Guided gradient class activation map (gGrad-CAM)

GGrad-CAM is a combination of Grad-CAM and GBP [99]. Grad-CAM is based on computing the gradient with respect to backpropagation till the final convolution layer, but it lacks the ability to extract fine-grained details. Therefore, GBP is used which computes a gradient using backpropagation over all the layers and therefore, provides more fine details. These two backpropagations work independently of each other, but are combined using a pointwise multiplication to obtain an attribution map.

#### Occlusion maps

Occlusion mapping is an XAI algorithm that (systematically) perturbates pixels/voxels with, for instance, a reference rectangle of the input image $f_{c}(I)$ and monitors how the feature maps and output prediction $y(x)$ changes using many computation intensive forward and backward propagations. From this a occlusion map $E_{Occ}(I,f)_{c}$ for a specific class can be calculated using linear regression (Equation 9).

$E_{Occ}(I,f)_{c}=\sum_{k} \frac{f_{c}\left( I \right)- f_{c}\left( I_{k}^{'} \right)}{m*m}$ Equation 9

#### Local Interpretable Model-Agnostic Explanations (LIME)

LIME works in an almost similar way as occlusion mapping [9]. However, instead of a predefined occlusion function, LIME perturbates super-pixels, which are a group of pixels that share common pixel/voxel characteristics. This creates interpretable local surrogate models that explains the models output prediction in a more data-driven way than previous described occlusion techniques.

#### SHapley Additive exPlanations (SHAP)

To calculate each feature attribution using a SHAP explanation model $g$ (Equation 10), also interaction between the features (coalitions) are considered, i.e. different permutations of coalitions can have different impact on model output. Therefore, to compute a SHAP value $\Phi_{j}\in R$for each feature $j$, all possible coalition $z^{'}\in\left\{ 0,1 \right\}^{M}$possibilities should be considered.

$g\left( z^{'} \right)=\Phi_{0}+\sum_{j=1}^{M} \Phi_{j}z_{j}^{'}$ Equation 10

KernelSHAP is an equivalent of LIME, apart from that the dependency of super-pixels is approximated using reference super-pixels values instead of non-informative super-pixels. Also, traditional LIME computes the weight of a feature according to the absolute distance to the original feature, i.e. the more absent super-pixels in the coalitions, the smaller the weight. SHAP, however, gives the highest weights to coalitions with sparse present super-pixels and to coalitions with many present super-pixels. This is because if a coalition consists of a single super pixel, we can learn about this super pixel’s isolated effect on the prediction. If a coalition consists of all but one super-pixel, we can learn about this super pixel’s total effect (interactions in coalitions).

Similar to KernelSHAP, DeepSHAP approximates the dependency of features using a selection of reference samples. However, feature contribution is approximated based on individual pixels/voxels instead of super-pixels. In comparison to traditional DeepLIFT, DeepSHAP differs by using a distribution of reference samples instead of a single reference value. Also, SHAP equations are used to linearize nonlinear components.

#### Explainable Deep Neural Network (xDNN)/prototyping

xDNN uses a prototype identification layer in the network to identify new data samples based on similarity to prototypes [78]. Prototypes (data clouds) are defined as local peaks identified by the density layers in the xDNN network. The density layer identifies new images by measuring the variance $\sigma$ and the Euclidian distance to the global mean $\mu$ of the images. Images $x_{i}$ that are closer to this global mean obtain a higher density value $D$, i.e. are more influenced by other images. Subsequently, the prototype layer assigns the new images based on these densities to the nearest (in the feature space) prototype using a nearest neighbour algorithm.

$D\left( x_{i} \right)=\frac{1}{1+\frac{\left\| x_{i}-\mu_{N} \right\|}{\sigma_{N}^{2}}}$ Equation 11

#### Capsule networks

CNNs have been seen as state-of-the-art approach to classify image data. It combines sets of features from multiple layers, extracting edges, shapes and objects. However, spatial relation between these features is lost and therefore CNNs are sensitive to spatial variation (orientation, scale, etc.) in the image data. Capsule networks, however, take the spatial relationship between features as an additional learning step [100]. Therefore, capsule networks have been seen more resilient to spatial variance and achieves significant higher accuracy than a CNN in multiple classification tasks. A capsule is a function that tries to describe the presence and the instantiation parameters (orientation, thickness, skewed, position, etc.) of a particular object (e.g. tumour, lungs, etc.) at a given location (Supplementary Figure 7). Similar as in a CNN, convolution layers extract features, but are than reshaped to compute a vector (magnitude and direction) for multiple objects. This vector (a probability) represent the object’s location and orientation throughout the capsule network, which in a traditional CNN is removed by using pooling layers. In a capsule network, the vectors from a lower capsule layer tries to predict the output for the higher layer based on the instantiation parameters, i.e. higher-level capsules detect combinations of these instantiation parameters. The predictions from the lower layer vectors are compared with each other and only the vectors with high agreement (small distance to mean of vectors) are routed to the following capsule layer (agreement by routing). This provides a less noisy and a more spatial orientated signal propagation throughout the network than a CNN. Also, these activations provide a more intuitive explanation of which feature belongs to which object, which is highly useful in XAI.

#### (Spatial) Attention mapping

The attention estimators can be added in current used CNNs and (can) consist of an average-pooling and a max-pooling operation to compute a comprehensive feature descriptor, followed by a convolution to generate a spatial attention map, which encodes where to emphasize or suppress local features. This encoding is performed by comparing the local feature vector with the global feature vector resulting in a compatibility score. A high compatibility score means that the local feature vector contains parts of the dominant image class and will be emphasized by the convolution layer. This way, local features which do not have high compatibility with the global vector are suppressed, resulting in less noisy and more target-specific signal propagation.

# Appendix B: Supplementary Figures

Supplementary Figure 1: Backpropagation over the ReLU layer in VG. From an activation layer $X_{n}$, the $X_{n}>0$ is computed and pointwise multiplied with the output reconstruction $R_{n+1}$ to create the following output reconstruction $R_{n}$.

Supplementary Figure 2: Backpropagation over the ReLU layer in DeconvNET. From a output reconstruction $R_{n+1}$, the $R_{n+1}>0$ is computed and pointwise multiplied with the same output reconstruction $R_{n+1}$to create the following output reconstruction R_n_.

Supplementary Figure 3: Backpropagation over the ReLU layer in GBP. From an activation layer $X_{n}$ and corresponding output reconstruction $R_{n+1}$, the $X_{n}>0$ and $R_{n+1}>0$ is computed, respectively. Subsequently, the $X_{n}>0$ and $R_{n+1}>0$ are pointwise multiplied with the same output reconstruction $R_{n+1}$ to create the following output reconstruction $R_{n}$.

Supplementary Figure 4: Layer-wise backpropagation over the neurons to compute the relevance scores ${{(R}_{n})}_{n}$ between each neuron.

Supplementary Figure 5: Instead of a gradient (A), DeepLIFT (B) calculates the slope for $f_{n}(x,y)$, which describes how y changes as x differs from $f_{0}(x,y)$. Therefore, although the gradient can be zero, DeepLIFT provides a non-zero value.

Supplementary Figure 6: From the weights $w_{c}^{n}$ corresponding to class $c$ for feature map $fn$, the CAM is calculated [3].

Supplementary Figure 7: Capsule Network uses groups of neurons (capsules) instead of neurons (single units). The output of each capsule is a vector (magnitude and direction) instead of a scaler (only magnitude) in case of a CNN, which are the instantiation parameters for objects (e.g. tumour) in the image. The instantiation parameters are not seen as individual objects, but as dependencies of each other. Using routing by agreement, capsules with high agreement between the lower and higher level capsules (red vectors) are emphasized, while the others are supressed.

# Appendix C: Supplementary Tables

Supplementary Table 1: Overview of the included studies

| **Overview of included literature** | | | | | |
| --- | --- | --- | --- | --- | --- |
| **AI** | **Patient cohort** | **Imaging modality** | **Data** | **Post-hoc/Ad-hoc** | **Study title** |
| Pretrained CNN | Cancer | CT | Imaging | Post-hoc | [2] |
| Unet | Cancer | MRI | Imaging | Post-hoc | [5] |
| Unet | Cancer | MRI | Imaging | Post-hoc | [14] |
| Pretrained CNN | Abdomen | CT | Imaging | Post-hoc | [15] |
| CNN | Ophthalmology | MRI | Imaging | Post-hoc | [16] |
| Unet | Neurology | MRI | Imaging | Post-hoc | [18] |
| CNN | Neurology | MRI | Imaging | Post-hoc | [20] |
| CNN | Cancer | MRI | Imaging | Post-hoc | [21] |
| CNN | Cardiology | CT | Imaging | Post-hoc | [23] |
| Attention estimator | COVID-19 | CT | Imaging | Ad-hoc and Post-hoc | [24] |
| Attention estimator | COVID-19 | CT | Imaging | Ad-hoc | [24] |
| Unet; CNN | Orthopaedic | MRI | Imaging | Post-hoc | [25] |
| CNN | Multiple sclerosis | MRI | Imaging | Post-hoc | [26] |
| Review | Cancer |  | Imaging | Ad-hoc and Post-hoc | [28] |
| Review | Review |  | Imaging | Ad-hoc and Post-hoc | [29] |
| CNN; ML | Cancer | CT | Imaging, radiomics and clinical | Post-hoc | [30] |
| CNN | Cancer | MRI | Imaging | Post-hoc | [31] |
| CNN | ADHD | fMRI | Imaging | Post-hoc | [32] |
| CNN | Cancer | MRI | Imaging | Post-hoc | [33] |
| Pretrained CNN | Cancer | MRI | Imaging | Post-hoc | [34] |
| Review | COVID-19 | CT | Imaging | Ad-hoc and Post-hoc | [35] |
| Pretrained CNN | COVID-19 | CT | Imaging | Post-hoc | [36] |
| CNN; Long short temporal memory | Lung | CT | Imaging | Post-hoc | [37] |
| Pretrained CNN | COVID-19 | CT | Imaging | Post-hoc | [38] |
| Pretrained CNN | COVID-19 | CT | Imaging | Post-hoc | [39] |
| CNN; ML | COVID-19 | CT | Imaging | Post-hoc | [40] |
| CNN | COVID-19 | CT | Imaging | Post-hoc | [41] |
| Unet; CNN | COVID-19 | CT | Imaging | Post-hoc | [42] |
| Pretrained CNN | COVID-19 | CT | Imaging | Post-hoc | [43] |
| CNN | COVID-19 | CT | Imaging | Post-hoc | [44] |
| Unet; CNN | COVID-19 | CT | Imaging | Post-hoc | [45] |
| CNN; MIL | Cancer | CT | Imaging | Post-hoc | [46] |
| CNN | COVID-19 | CT | Imaging | Post-hoc | [47] |
| CNN | Stroke | MRI | Imaging | Post-hoc | [48] |
| CNN | Stroke | CT | Imaging | Post-hoc | [49] |
| CNN | Cardiology | PET | Imaging and clinical | Post-hoc | [50] |
| Generative adversarial network; CNN | Alzheimer | MRI | Imaging | Post-hoc | [51] |
| CNN | Schizophrenia | MRI | Imaging | Post-hoc | [52] |
| Pretrained CNN | Cancer | CT | Imaging | Post-hoc | [53] |
| Attention estimator | Alzheimer | MRI | Imaging | Post-hoc | [54] |
| Pretrained CNN | Cancer | MRI | Imaging | Post-hoc | [55] |
| CNN | Tuberculosis | CT | Imaging | Post-hoc | [56] |
| CNN | Cancer | MRI | Imaging | Post-hoc | [57] |
| Pretrained CNN | Alzheimer | MRI | imaging | Post-hoc | [58] |
| CNN | Frontotemporal dementia | MRI | Imaging | Post-hoc | [60] |
| CNN | Schizophrenia | fMRI | Imaging | Post-hoc | [61] |
| Pretrained CNN | Artefact | CT | Imaging | Post-hoc | [62] |
| Attention estimator | Artefact | MRI | Imaging | Ad-hoc | [63] |
| Unet; CNN | Cancer | MRI | Imaging | Post-hoc | [64] |
| CNN | Alzheimer | MRI | Imaging | Post-hoc | [65] |
| CNN | Cancer | MRI | Imaging | Post-hoc | [67] |
| Pretrained CNN | COVID-19 | CT | Imaging | Post-hoc | [68] |
| Pretrained CNN | COVID-19 | CT | Imaging | Post-hoc | [69] |
| CNN | Cancer | MRI | Imaging | Post-hoc | [71] |
| Unet | Cancer | MRI | Imaging | Post-hoc | [72] |
| Unet; Support vector machine (SVM) | Cancer | MRI | Imaging and clinical | Post-hoc | [73] |
| Unet; Neural Network | Orthopaedic | MRI | Imaging | Post-hoc | [74] |
| Pretrained CNN | COVID-19 | CT | Imaging | Post-hoc | [75] |
| CNN | Cancer | CT | Imaging | Post-hoc | [76] |
| Attention estimator; Regression | Cancer | CT | Imaging | Ad-hoc | [77] |
| xDNN | COVID-19 | CT | Imaging | Ad-hoc | [81] |
| xDNN | COVID-19 | CT | Imaging | Ad-hoc | [82] |
| xDNN | COVID-19 | CT | Imaging | Post-hoc | [83] |
| xDNN; Attention estimator | COVID-19 | CT | Imaging | Ad-hoc | [83] |
| xDNN | COVID-19 | CT | Imaging | Ad-hoc | [84] |
| Capsule Network | Cancer | CT | Imaging | Ad-hoc | [86] |
| Attention estimator | Alzheimer | MRI | Imaging | Ad-hoc | [88] |
| Attention estimator | COVID-19 | CT | Imaging | Ad-hoc | [89] |
| Attention estimator | Cancer | CT | Imaging | Ad-hoc | [90] |
| Attention estimator | Cancer | CT | Imaging and radiomics | Ad-hoc | [91] |
| Attention estimator; Unet | Fetal | MRI | Imaging | Ad-hoc | [92] |
| Attention estimator; MIL | COVID-19 | CT | Imaging | Ad-hoc | [93] |
| Attention estimator; MIL | COVID-19 | CT | Imaging | Ad-hoc | [94] |
| Attention estimator; MIL | Cancer | CT | Imaging | Ad-hoc | [95] |
| Attention estimator; GRU | COVID-19 | CT | Imaging | Ad-hoc | [96] |
|  |  |  |  |  |  |
|  | Pulmonology: n=27  Oncology: n=24  Neurology: n=15  Artefact: n=2  Orthopaedic: n=2  Cardiology: n=2  Review: n=2  Ophthalmology: n=1 | CT: n=41  MRI: n=29  fMRI: n=2  PET: n=1 | Imaging: n=75  Imaging and clinical: n=2  Imaging and radiomics: n=1  Imaging, clinical and radiomics: n=1 | Post-hoc: n=54  Ad-hoc: n=17  Both: n=4 |  |

Supplementary Table 2: Search strategy in PubMed

| **Search** | **Query** | **Results** |
| --- | --- | --- |
| **#6** | **#5 NOT ("Animals"[Mesh] NOT "Humans"[Mesh])** | **503** |
| **#5** | **#4 NOT OCT[tiab]** | **513** |
| **#4** | **#1 AND #2 AND #3** | **535** |
| **#3** | **"Multimodal Imaging"[Mesh] OR "Neuroimaging"[Mesh] OR "Radiography"[Mesh] OR "Radionuclide Imaging"[Mesh] OR "Tomography, X-Ray"[Mesh] OR "Magnetic Resonance Imaging"[Mesh] OR "Whole Body Imaging"[Mesh] OR imaging*[tiab] OR magnetic-resonance*[tiab] OR Tomograph*[tiab] OR CT-scan*[tiab] OR CAT-scan*[tiab] OR CT-X-Ray*[tiab] OR CT-XRay*[tiab] OR PET-CT[tiab] OR PET-Scan[tiab] OR positron[tiab] OR emission[tiab] OR scintigra*[tiab] OR SPECT[tiab]** | **2,721,554** |
| **#2** | **"Artificial Intelligence"[Mesh] OR "Bayes Theorem"[Mesh] OR "Markov Chains"[Mesh] OR "Latent Class Analysis"[Mesh] OR AdaBoost[tiab] OR AI[tiab] OR Artificial-Intelligence*[tiab] OR autoencoder*[tiab] OR auto-encoder*[tiab] OR Automatic-segmentation*[tiab] OR Back-propagation*[tiab] OR Bayesian-learning[tiab] OR gradient-boosting[tiab] OR classification-algorithm*[tiab] OR Computational-Intelligen*[tiab] OR Computer-heuristic*[tiab] OR Computer-reasoning*[tiab] OR Computer-vision*[tiab] OR Connectionist-model*[tiab] OR deep-belief-network*[tiab] OR Deep-learning[tiab] OR generalized-additive-model*[tiab] OR generalised-additive-model*[tiab] OR Heuristic-learning[tiab] OR Hierarchical-learning*[tiab] OR Image-Interpretation*[tiab] OR Image-recognition[tiab] OR Kernel-method*[tiab] OR latent-process*[tiab] OR latent-variable*[tiab] OR Learning-algorithm*[tiab] OR learning-automata[tiab] OR learning-machine*[tiab] OR Neural-network*[tiab] OR perceptron[tiab] OR Qlearning[tiab] OR Q-learning[tiab] OR reinforcement-learning[tiab] OR Rule-based[tiab] OR self-organising-map*[tiab] OR Supervised-learning[tiab] OR Transfer-learning[tiab] OR Unsupervised-learning[tiab] OR XAI[tiab]** | **347,802** |
| **#1** | **Explainable[tiab] OR Interpretable[tiab] OR XAI[tiab]** | **12,719** |

Supplementary Table 3: Search strategy in Embase.com

| **Search** | **Query** | **Results** |
| --- | --- | --- |
| **#7** | #6 NOT oct:ti,ab,kw | **464** |
| **#6** | #5 NOT ([animals]/lim NOT [humans]/lim) | **484** |
| **#5** | #4 NOT ('chapter'/it OR 'conference abstract'/it OR 'conference review'/it OR 'editorial'/it OR 'note'/it) | **498** |
| **#4** | #1 AND #2 AND #3 | **589** |
| **#3** | 'multimodal imaging'/exp OR 'neuroimaging'/exp OR 'radiography'/exp OR 'radioisotope diagnosis'/exp OR 'tomography'/de OR 'brain tomography'/exp OR 'computer assisted tomography'/exp OR 'digital breast tomosynthesis'/exp OR 'emission tomography'/exp OR 'kidney tomography'/exp OR 'nuclear magnetic resonance imaging'/exp OR 'whole body tomography'/exp OR 'x-ray tomography'/exp OR (imaging* OR magnetic-resonance* OR Tomograph* OR CT-scan* OR CAT-scan* OR CT-X-Ray* OR CT-XRay* OR PET-CT OR PET-Scan OR positron OR emission OR scintigra* OR SPECT):ti,ab,kw | **4,038,032** |
| **#2** | 'artificial intelligence'/exp OR 'Bayes theorem'/exp OR 'stochastic model'/exp OR 'latent structure analysis'/exp OR (AdaBoost OR AI OR Artificial-Intelligence* OR autoencoder* OR auto-encoder* OR Automatic-segmentation* OR Back-propagation* OR Bayesian-learning OR gradient-boosting OR classification-algorithm* OR Computational-Intelligen* OR Computer-heuristic* OR Computer-reasoning* OR Computer-vision* OR Connectionist-model* OR deep-belief-network* OR Deep-learning OR generalized-additive-model* OR generalised-additive-model* OR Heuristic-learning OR Hierarchical-learning* OR Image-Interpretation* OR Image-recognition OR Kernel-method* OR latent-process* OR latent-variable* OR Learning-algorithm* OR learning-automata OR learning-machine* OR Neural-network* OR perceptron OR Qlearning OR Q-learning OR reinforcement-learning OR Rule-based OR self-organising-map* OR Supervised-learning OR Transfer-learning OR Unsupervised-learning OR XAI):ti,ab,kw | **357,210** |
| **#1** | (Explainable OR Interpretable OR XAI):ti,ab,kw | **15,454** |

Supplementary Table 4: Search strategy in Clarivate Analytics/Web of Science Core Collection

| **Search** | **Query** | **Results** |
| --- | --- | --- |
| **#5** | #4 NOT TS=(oct) | **535** |
| **#4** | #1 AND #2 AND #3 | **558** |
| **#3** | TS=(imaging* OR magnetic-resonance* OR Tomograph* OR CT-scan* OR CAT-scan* OR CT-X-Ray* OR CT-XRay* OR PET-CT OR PET-Scan OR positron OR emission OR scintigra* OR SPECT) | **3,039,793** |
| **#2** | TS=(AdaBoost OR AI OR Artificial-Intelligence* OR autoencoder* OR auto-encoder* OR Automatic-segmentation* OR Back-propagation* OR Bayesian-learning OR gradient-boosting OR classification-algorithm* OR Computational-Intelligen* OR Computer-heuristic* OR Computer-reasoning* OR Computer-vision* OR Connectionist-model* OR deep-belief-network* OR Deep-learning OR generalized-additive-model* OR generalised-additive-model* OR Heuristic-learning OR Hierarchical-learning* OR Image-Interpretation* OR Image-recognition OR Kernel-method* OR latent-process* OR latent-variable* OR Learning-algorithm* OR learning-automata OR learning-machine* OR Neural-network* OR perceptron OR Qlearning OR Q-learning OR reinforcement-learning OR Rule-based OR self-organising-map* OR Supervised-learning OR Transfer-learning OR Unsupervised-learning OR XAI) | **667,038** |
| **#1** | TS=(Explainable OR Interpretable OR XAI) | **22,147** |

# References

[9] Ribeiro M. T., Singh S., Guestrin C. “Why should I trust you?”: explaining the predictions of any classifier. in Proceedings of the 22nd ACM SIGKDD International Conference on Knowledge Discovery and Data Mining (2016).

[19] Springenberg J., Dosovitskiy A., Brox T., Riedmiller M. Striving for simplicity: the all convolutional net. arXiv:1412.6806 (2014). doi: 10.48550/arXiv.1412.6806

[78] Yang Z, Hu Z, Ji H, Lafata K, Vaios E, Floyd S, et al. A neural ordinary differential equation model for visualizing deep neural network behaviors in multi‐parametric MRI‐based glioma segmentation. Med Phys. (2023):1–14. doi: 10.1002/mp.16286

[98] Simonyan K., Vedaldi A., Zisserman A. Deep inside convolutional networks: visualising image classification models and saliency maps. arXiv:1312.6034. (2013). doi: doi: 10.48550/arXiv.1312.6034

[99] Selvaraju R. R., Cogswell M., Das A., Vedantam R., Parikh D., Batra D. Grad-CAM: visual explanations from deep networks via gradient-based localization. in 2017 IEEE International Conference on Computer Vision (ICCV), 2017 Oct 22–29 (2017)

[100] Sabour S., Frosst N., Hinton G. Dynamic routing between capsules. arXiv.1710.09829 (2017). doi: 10.48550/arXiv.1710.09829
