# Supplementary figures and images for: Explainable artificial intelligence (XAI) in radiology and nuclear medicine: a literature review
Source: Front Med (Lausanne). 2023 May 12;10:1180773. doi: 10.3389/fmed.2023.1180773 (PMC10213317; doi:10.3389/fmed.2023.1180773)

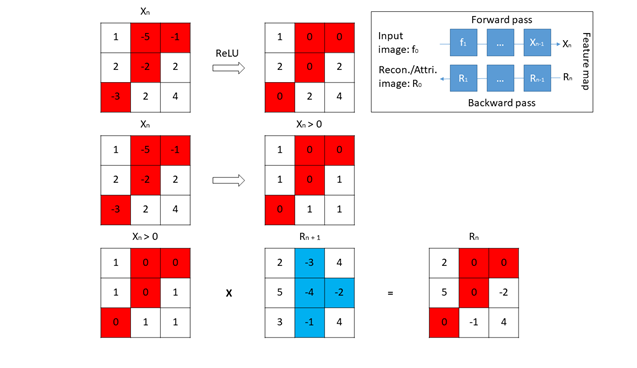

Supplement: Supplementary file 2 [file Image_1.tif]

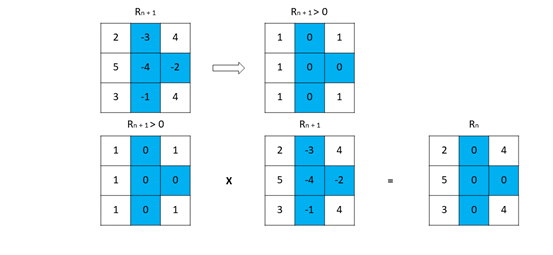

Supplement: Supplementary file 3 [file Image_2.tif]

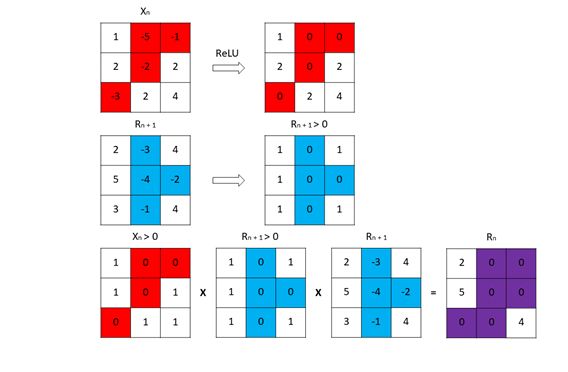

Supplement: Supplementary file 4 [file Image_3.tif]

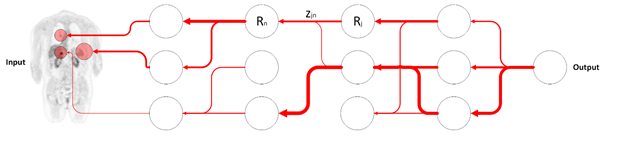

Supplement: Supplementary file 5 [file Image_4.tif]

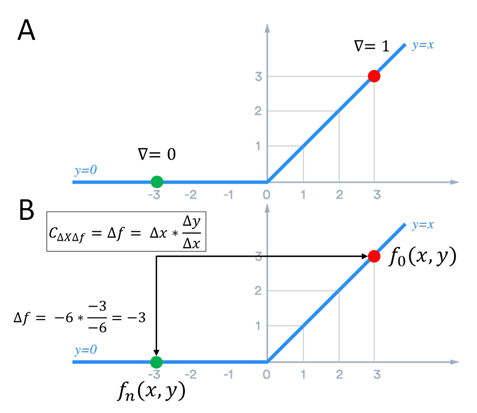

Supplement: Supplementary file 6 [file Image_5.tif]

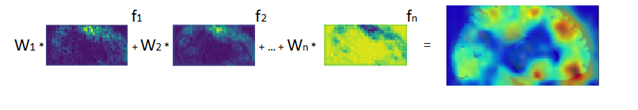

Supplement: Supplementary file 7 [file Image_6.tif]

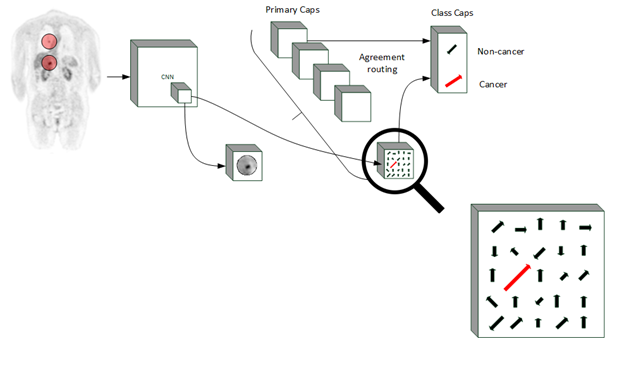

Supplement: Supplementary file 8 [file Image_7.tif]
